# Breast Cancer Screening Participation and Internet Search Activity in a Japanese Population: Decade-Long Time-Series Study

**DOI:** 10.2196/64020

**Published:** 2025-03-04

**Authors:** Noriaki Takahashi, Mutsuhiro Nakao, Tomio Nakayama, Tsutomu Yamazaki

**Affiliations:** ^1^Graduate School of Medicine, International University of Health and Welfare, Tokyo, Japan; 2 Division of Screening Assessment and Management, National Cancer Center Institute for Cancer Control, Tokyo, Japan; 3 Institute of Stress Management, Showa University, Tokyo, Japan; 4 Innovation and Research Support Center, International University of Health and Welfare, Tokyo, Japan

**Keywords:** breast cancer, cancer screening, internet use, mass media, public health surveillance, health belief model, mammography, awareness, Japanese, Google

## Abstract

**Background:**

Breast cancer is a major health concern in various countries. Routine mammography screening has been shown to reduce breast cancer mortality, and Japan has set national targets to improve screening participation and increase public attention. However, collecting nationwide data on public attention and activity is not easy. Google Trends can reveal changes in societal interest, yet there are no reports on the relationship between internet search volume and nationwide participation rates in Japan.

**Objective:**

This study aims to reveal and discuss the relationship between public awareness and actual behavior in breast cancer screening by examining trends in internet search volume for the keyword “breast cancer screening” and participation rates over a decade-long period.

**Methods:**

This time-series study evaluated the association between internet search volume and breast cancer screening participation behavior among women aged 60‐69 years in Japan from 2009 to 2019. Relative search volume (RSV) data for the search term “breast cancer screening (nyuugan-kenshin)” were extracted from Google Trends as internet search volume. Breast cancer screening and further assessment participation rates were based on government municipal screening data. Joinpoint regression analyses were conducted with weighted BIC to evaluate the time trends. An ethics review was not required because all data were open.

**Results:**

The RSV for “breast cancer screening (nyuugan-kenshin)” peaked in June 2017 (100) and showed clear spikes in June 2016 (94), September (69), and October (77) 2015. No RSVs above 60 were observed except around these three specific periods, and the average RSV for the entire period was 30.7 (SD 16.2). Two statistically significant joinpoints were detected, rising in December 2013 and falling in June 2017. Screening participation rates showed a temporary increase in 2015 in a slowly decreasing trend, and no joinpoints were detected. Further assessment participation rates showed a temporary spike in 2015 in the middle of an increasing trend, with a statistically significant point of slowing increase detected in 2015. Post hoc manual searches revealed that Japanese celebrities’ breast cancer diagnoses were announced on the relevant dates, and many Japanese media reports were found.

**Conclusions:**

This study found a notable association between internet search activity and celebrity cancer media reports and a temporal association with screening participation in breast cancer screening in Japan. Celebrity cancer media reports triggered internet searches for cancer screening, but this did not lead to long-term changes in screening participation behavior. This finding suggests what information needs to be provided to citizens to encourage participation in screening.

## Introduction

Breast cancer is a major health concern that affects large numbers of women in various countries. It is the most common cancer in women, with approximately 2.3 million new cases diagnosed and 680,000 deaths reported in 2020 [1,2]. The disease burden of breast cancer is also high in Japan. The age-adjusted incidence rate of breast cancer continues to increase every year, and it has been reported that breast cancer accounts for about 20% of all cancers in women in Japan [3,4]. As Japan’s population ages, the burden of breast cancer is predicted to increase [5].

Several studies have indicated the efficacy of mammography screening for breast cancer in reducing the burden of breast cancer. Routine mammography screening has been shown to reduce breast cancer mortality by 25%‐31% [6,7]. The long-term effects of mammography have also been shown in a 30-year follow-up study [8]. In Japan, national policy recommends biennial mammography for breast cancer screening in women older than 40 years [9]. Therefore, screening is a practical approach to reducing the long-term burden of breast cancer, and its importance is increasing in Japan.

Cancer screening programs in Japan are divided into two main types: municipal screening and workplace screening. Municipalities conduct screenings, and information is collected by the Ministry of Health, Labor, and Welfare and made available to the public. This is the only data on cancer screening for all regions in Japan for which the government reports actual statistics annually. This report includes data on screening and further assessment participation. The participation rate is the proportion of the target population that receives the primary screening test, which in the case of breast cancer screening is mammography. The further assessment participation rate is the proportion of women who, after a positive screening test result, receive the following test to confirm the diagnosis: fine needle aspiration cytology or core needle biopsy in breast cancer screening. Even if a person participates in screening, the effectiveness of cancer screening will not be fully realized unless the screening-positive person receives a further assessment. The further assessment participation rate is an important indicator, as is the screening participation rate. Japan has set national targets to improve screening participation, further assessment participation, and increase public attention to the importance of cancer screening [10]. Understanding the public’s attention and behavior around cancer screening is critical to improving screening participation rates. However, it is not easy to collect nationwide data on public attention and activity to assess the association with screening participation.

Internet search volume has recently become one of the most valuable tools for exploring human interests and behavior. Google Trends is a popular open web-based tool that quantifies changes in internet search volume for a given term based on actual Google search history [11,12]. Google Trends is used for academic research in fields as diverse as social science, economics, language, and medicine and can also reveal changes in societal interest in public health issues [13]. Google Trends initially focused on detecting infectious disease outbreaks, and past studies have reported early detection of influenza outbreaks [14].

Google Trends is now expected to be used in noncommunicable disease areas such as mental health and preventive behaviors and is a potential source of information for understanding the public’s interest in and behaviors around cancer screening [15]. Malaysia reported a significant correlation between Google Trends search patterns and Pink Ribbon Month, a breast cancer awareness campaign [16]. Among several internet search engines, Google was also shown to have the best search validity (regarding whether a web page could be opened) for breast cancer screening information [17]. In contrast, a previous Japanese study analyzed trends in cervical cancer and reported no change in public interest during the cervical cancer awareness month [18].

Therefore, there is considerable interest in the relationship between internet search activity and cancer screening. In Japan, it would be valuable to determine the relationship between public attention to cancer screening and participation rates at the national level to understand public awareness and behavior. However, there are no reports on the relationship between changes in internet search volume and long-term trends in nationwide participation rates in Japan.

This study examined the relationship between public awareness and actual behavior in breast cancer screening at the national level. This study is the first report in Japan to reveal and discuss the background of the relationship between trends in internet search volume for the keyword “breast cancer screening” and participation rates over a decade-long period. As an example of the application of epidemiologic research using internet search volume, this approach could provide knowledge for promoting cancer screening and providing appropriate information.

## Methods

### Study Design

This time-series study uses internet search volume and national cancer screening statistics. Internet search volume targets those who conducted searches in Japanese using Google in Japan. Cancer screening data targets municipal screening in Japan. Both data are openly available on the web.

### Data Sources (Internet Search Volume)

Google Trends is a data tool that publishes the volume of keyword searches worldwide in Google Search, an internet search engine, since 2004. This tool allows users to access the relative search volume (RSV) but not the absolute number of searches. RSV is calculated on a scale of 0 to 100 based on the volume with the most searches per unit of time in the defined region, period, category, and search term. For example, RSV=30 means 30% of the highest search volume observed within a given condition. RSVs can assess changes in interest in a particular term by showing the relative value of search volume trends over time.

The search term was “nyuugan-kenshin,” which means “breast cancer screening” in Japanese. Monthly RSV data from the Google Trends platform were retrieved on September 17, 2023. Since the Japanese term “nyuugan-kenshin” is written as one continuous word without any spaces, we did not enclose it in quotes when using it in Google Trends. Because Google Trends does not provide a “Topic” option for the Japanese term “nyuugan-kenshin,” we used the “Search Term” option instead. It was set to Japan as the target region and 2009‐2019 as the target period. To ensure that all possible contexts in which the term might appear were captured, we set “All categories” as the “Category” and “Web Search” as the “Search Type.”

If a significant trend increase was observed, a post hoc manual search was conducted using the “Related Keywords” feature of Google Trends to see if any socially essential media reports might be related to the increase.

### Data Sources (Cancer Screening)

For cancer screening, this study included screening participation rates and further assessment participation rates in municipal screening for breast cancer in Japan from 2009 to 2019. Municipal screening does not include individuals who participate in workplace screening. Consequently, when calculating screening participation rates for ages 40 years and older using the population as the denominator, workplace screening participants are excluded from the numerator. This omission can lead to fluctuations in time-series data, for example, if there is a change over time in the proportion of working individuals. Furthermore, when participation rates differ by age group, they are also affected by changes in the age distribution over time. To eliminate this effect as much as possible and to improve the time-series analysis’s validity, the calculation of participation rates was restricted to women aged 60‐69, who are mainly retired. The number of participants in screening, positive cases in screening, and further assessment participants were obtained from the “Report on Regional Public Health Services and Health Promotion Services” by the Ministry of Health, Labor, and Welfare [19]. Population data were obtained from the Statistics Bureau of the Ministry of Internal Affairs and Communications [20]. Screening participation rates were calculated by dividing the number of screening participants by the population of women in the target age group. The further assessment participation rates were calculated by dividing the number of further assessment participants by the number of positive screening cases. The recommended interval for breast cancer screening in Japan is once every two years, and the original “screening participation rates” are calculated by considering the number of participants screened for two years. However, this calculation method equalizes two years of information and may not detect sensitive value changes. As this study aimed to detect changes over time rather than absolute assessments, “screening participation rates” were defined as calculated values per one-year period and used in the analyses.

### Statistical Analysis

Joinpoint regression analyses were performed to assess RSV trends quantitatively. This analysis is an appropriate way to examine data over time and statistically detect points of change in gradient [21]. The software used was the Joinpoint Regression Program (version 5.0.2, Statistical Research and Applications Branch, National Cancer Institute) [22]. The statistical method used for joinpoint detection was weighted BIC, a standard method in Program version 5.0 and later. Weighted BIC is the most flexible and adaptable method for various situations in this software. Joinpoint regression analysis requires many computing resources, and the calculation time depends on the maximum number of detectable joinpoints set before the calculation. This analysis’s maximum number of joinpoints was set to 3 due to calculation time. Changing the maximum number of joinpoints can alter the significance level for individual tests and potentially change the number of joinpoints in the optimal model [22]. When no joinpoints were detected in the initial analysis, we conducted additional analyses with the maximum number set to two and one. The statistical significance level for joinpoint detection was set to 0.05.

### Ethical Considerations

This study was conducted per the principles of the Declaration of Helsinki. An ethics review was not required because all data used in this study were open. For this type of study, formal consent is not required.

## Results

### Internet Search Volume

**Figure 1.**
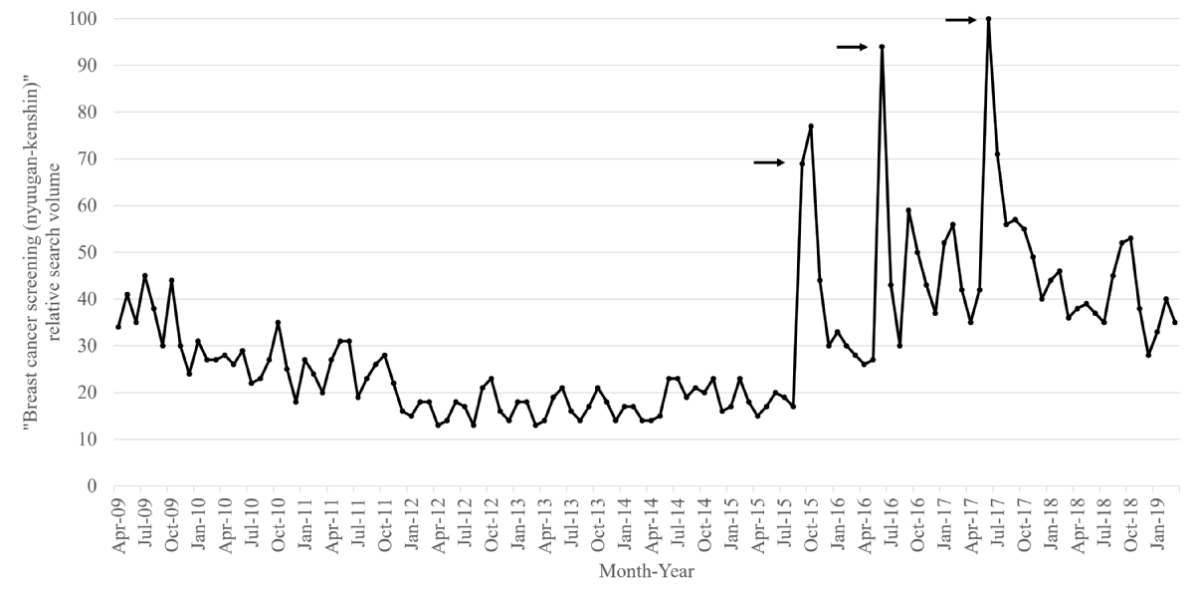
Monthly “breast cancer screening (nyuugan-kenshin)” relative search volumes in Japan from 2009 to 2019, based on Google Trends. The black arrows show the timing of media reports on the celebrities’ breast cancer diagnoses or passing.

**Figure 2.**
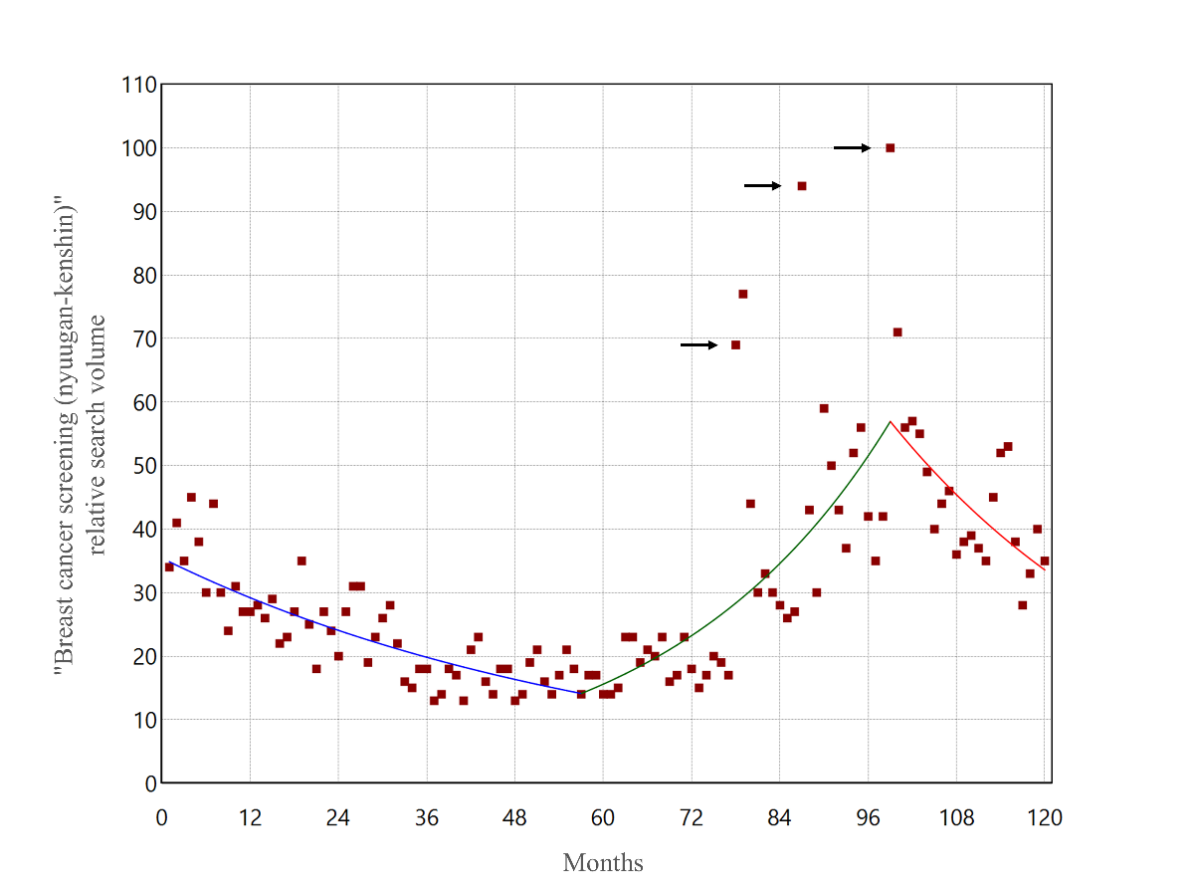
Joinpoint regression analysis of the monthly “breast cancer screening (nyuugan-kenshin)” relative search volumes in Japan from 2009 to 2019. Two significant joinpoints were detected (December 2013 and June 2017). The black arrows show the timing of media reports on the celebrities’ breast cancer diagnoses or passing.

Figure 1 shows the trend of RSVs for the search term “breast cancer screening (nyuugan-kenshin)” from 2009 to 2019. The RSV peaked in June 2017 (100) and showed clear spikes in June 2016 (94), September 2015 (69), and October 2015 (77). No RSVs above 60 were observed except around these three specific periods. The average RSV for the entire period was 30.7 (SD 16.2). Figure 2 shows the results of the joinpoint regression analysis for RSVs. Two statistically significant joinpoints were detected, rising in December 2013 and falling in June 2017.

### Cancer Screening Participation

**Figure 3.**
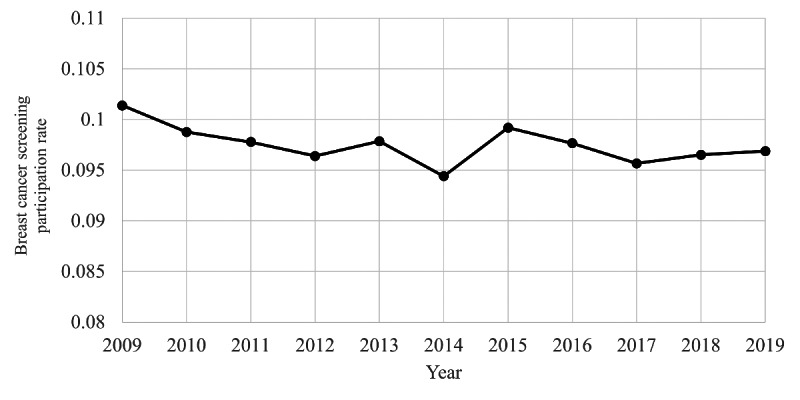
Annual breast cancer screening participation rates (mammography) among Japanese women aged 60‐69 years from 2009 to 2019, based on municipal screening data. The rate is the proportion of screening participants in the target population.

**Figure 4.**
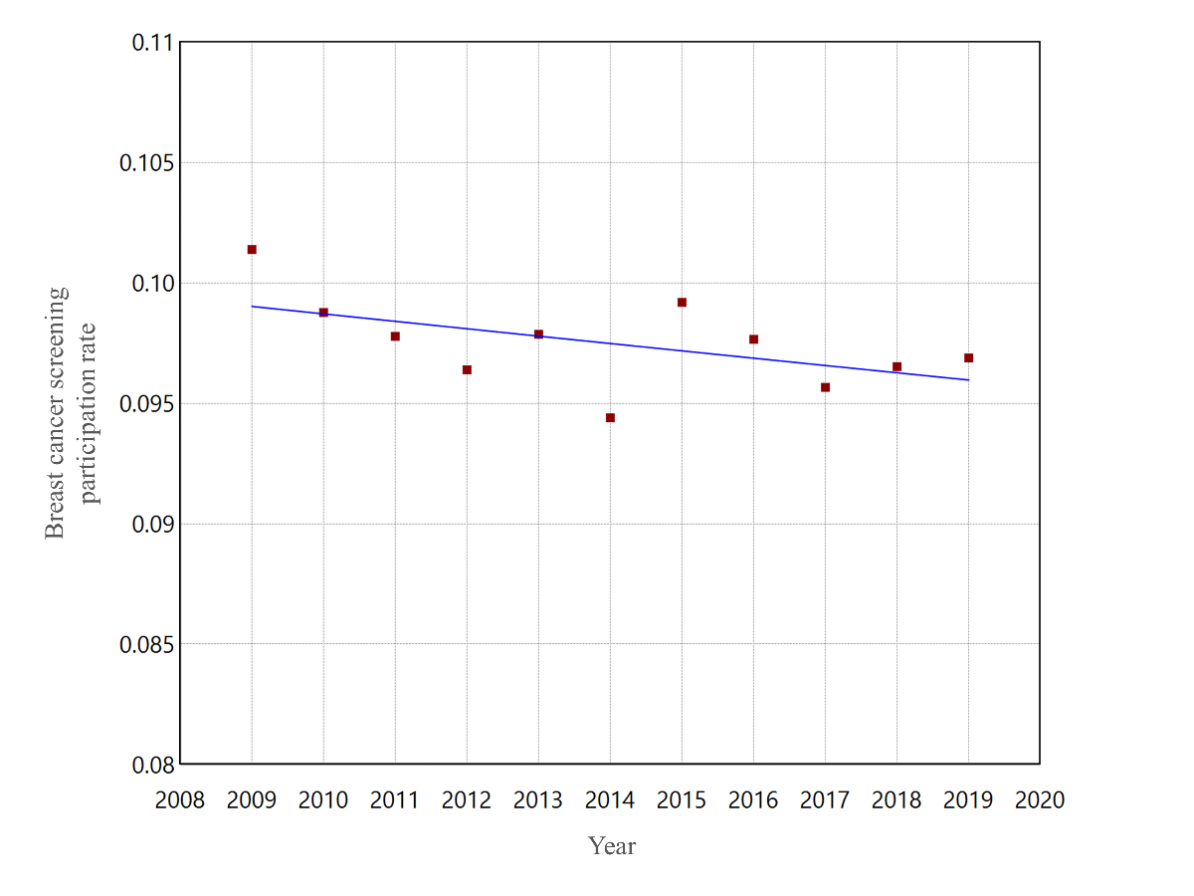
Joinpoint regression analysis of the annual breast cancer screening participation rates (mammography) among Japanese women aged 60‐69 years from 2009 to 2019. No joinpoints were detected.

Figure 3 shows the trend of breast cancer screening participation rates from 2009 to 2019. Visual observation shows a temporary increase in 2015 in the slowly decreasing trend. Figure 4 shows the results of the joinpoint analysis for screening participation rates. No joinpoints were detected. Even in additional analyses with the maximum number set to two or one, no joinpoints were detected.

**Figure 5.**
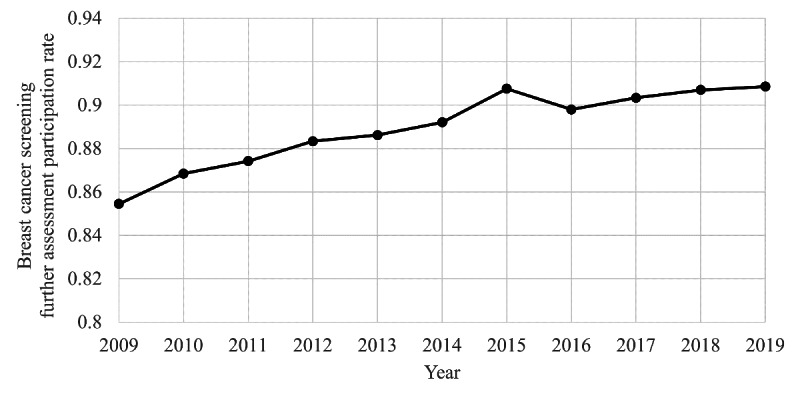
Annual breast cancer screening further assessment participation rates among Japanese women aged 60‐69 years from 2009 to 2019, based on municipal screening data. The rate is the proportion of women who received fine needle aspiration cytology or core needle biopsy following a positive screening result.

**Figure 6.**
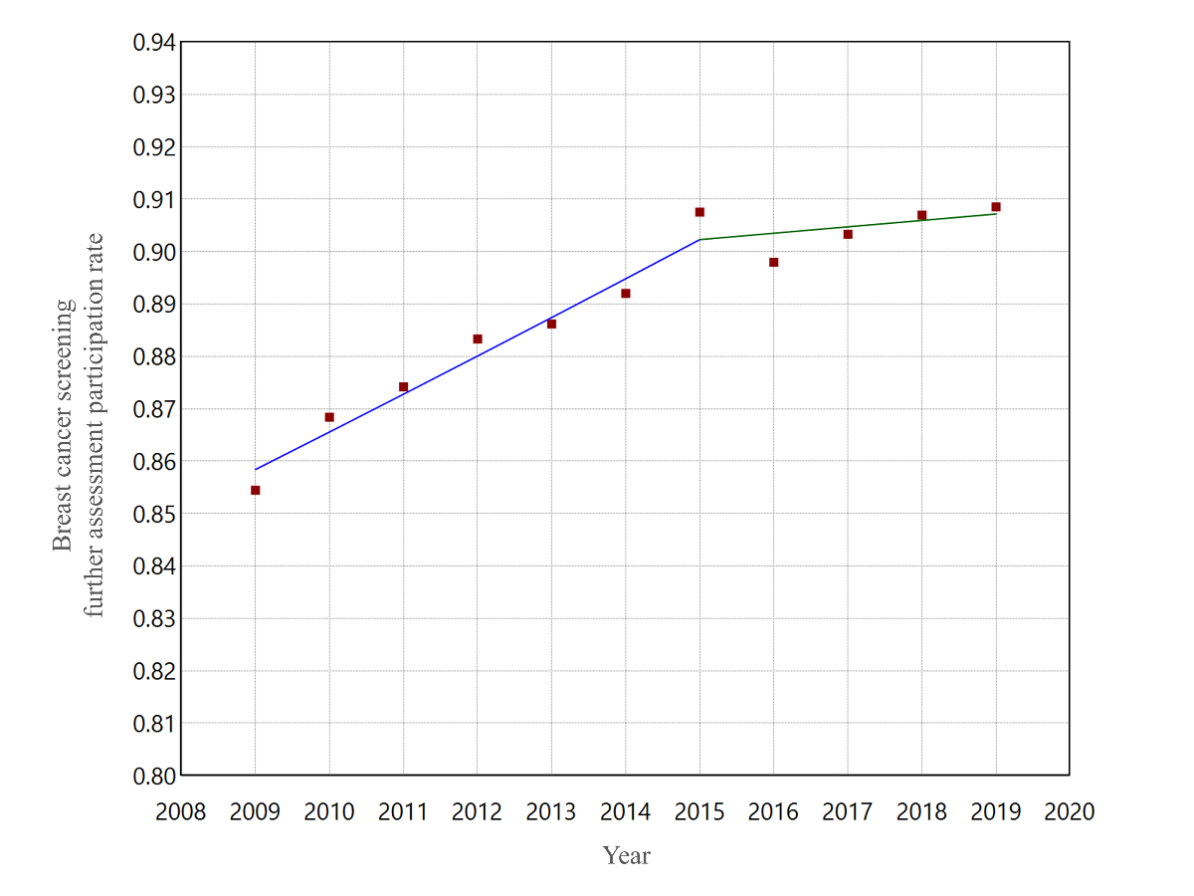
Joinpoint regression analysis of the annual breast cancer screening further assessment participation rates among Japanese women aged 60‐69 years from 2009 to 2019. One significant joinpoint was detected in 2015.

Figure 5 shows the trend of further assessment participation rates for breast cancer screening from 2009 to 2019. Visual observation shows a temporary spike in 2015 in the middle of an increasing trend. Figure 6 shows the result of the joinpoint analysis for further assessment participation rates. While the trend has been increasing for the entire period, a statistically significant point of slowing increase was detected in 2015. The year 2015 was the maximum for screening and further assessment participation rates, except for 2009 and 2019, the two ends of the period covered.

### Post Hoc Manual Search

When RSV spiked in 2015, 2016, and 2017, three periods were explored post hoc. A detailed review of the RSV data downloaded from Google Trends, restricting the period specified, revealed a notable spike on September 23 and 24, 2015, June 9, 2016, and June 23, 2017. The day’s news and Google Trends “Related Keywords” were manually searched for these three periods. For the term “nyuugan-kenshin,” only general keywords such as “examination,” “cost,” “mammography,” “breastfeeding,” and “symptoms” were suggested on each of the relevant dates. In contrast, as “Related Keywords” for “nyuugan (breast cancer)” on September 23 and 24, 2015, a Japanese celebrity, AH, was suggested as a nonmedical term. A Google search limited to the same period revealed that her breast cancer incidence was announced on September 23, 2015, and many Japanese media reports were found. In the same way, the Japanese celebrity MK’s breast cancer incidence was announced on June 9, 2016, and her passing on June 23, 2017, confirmed many media reports.

## Discussion

### General Interpretation of the Results

We conducted a time-series study for 2009‐2019 on Japanese internet search volume and breast cancer screening data for 60‐69-year-olds. Internet search RSVs for “breast cancer screening” spiked notably in three specific periods in September 2015, June 2016, and June 2017. The joinpoint analysis for RSVs revealed two joinpoints in December 2013 and June 2017, showing an increase over the period, including the spikes mentioned above. The 10-year trend in internet searches for breast cancer screening was dynamically changing, down, up, and down. The joinpoint of screening data was not detected for screening participation rates but was detected in 2015 for further assessment. Upon examination of Figure 5, it might be reasonable to interpret this as a temporary increase in 2015 and a return to the original trend from 2016 onwards. Indeed, the screening and further assessment participation rates reached their maximum in 2015, except for 2009 and 2019, the two ends of the period covered. A post hoc search for the timing of the three RSV spikes from 2015 to 2017 was consistent with the dates of media reports of breast cancer incidences and the passing of Japanese celebrities.

It is worth noting that 2015 marked the timing of the first media reports on celebrities, the first notable increase in RSVs, and the short-term maximum in the screening and further assessment participation rates. In particular, the consistency of the three dates between celebrity media reports and search trends is evident. RSV provided by Google Trends is not an absolute number of searches but a relative measure of the maximum number of searches scaled to 100 within a defined period. Therefore, if there is even one moment of drastic increase in search, RSV for the rest of the period will be relatively low. The fact that the average volume for the entire period in this analysis was 30.7 (SD 16.2) highlights the magnitude of the three spikes. Many individuals became interested in breast cancer screening when AH and MK were featured in the media, which probably triggered internet search behavior. Additionally, screening and further assessment participation rates increased temporarily in 2015, suggesting some individuals may have engaged in screening participation behaviors. Celebrity media reports may have influenced individuals, leading to search and screening participation behavior. This contrasts with a previous Japanese study that showed no association between cervical cancer awareness months and RSVs for “cervical cancer” [18].

However, screening and further assessment participation rates showed only a temporary spike in 2015 and did not increase the long-term trend. RSVs also declined after the 2017 joinpoint. These findings provide insight into the mechanisms necessary for citizens to be concerned about, act on, and maintain their health. To interpret this study’s results, referring to the findings of established behavioral models and previous studies would be appropriate.

### Interpretation Based on the Health Belief Model

Participation behavior in cancer screening has been a critical subject of study in the Health Belief Model (HBM). HBM is a theoretical model in the behavioral sciences that aims to explain, predict, and promote individual health behaviors. This model was developed in the 1950s to understand the factors determining participation in immunization and screening [23-25]. HBM considers that individual health behaviors are determined by the interaction of six factors: “perceived susceptibility,” “perceived severity,” “perceived benefits,” “perceived barriers,” “self-efficacy,” and “cues to action.” The model is widely used to design education and promotion programs for health activities. In cancer screening, the model has been primarily used to improve participation in colorectal cancer screening and has been validated in several randomized controlled trials [26,27]. Recently, there has been much research on breast cancer screening [28-33].

It would be meaningful to interpret the results of this study based on HBM. In the media reports, AH was 48 years old, and MK was 32 when they were diagnosed. Consecutive media reports of breast cancer in young celebrities may have caused “perceived susceptibility” among the public. MK passed away about a year after her diagnosis was announced. The sad outcome of the celebrity, which had been worrying through media reports, would have caused “perceived severity” for the public. Indeed, the RSV peaked on June 23, 2017, when MK’s passing was announced. MK’s weblog about her fight against breast cancer has attracted attention in Japan and around the world, and she was named one of the BBC’s 100 Women of the Year in 2016 [34,35]. Citizen exposure to a series of media reports may have fulfilled these elements in the HBM. Additionally, for those who were already aware of “susceptibility” and “severity,” media reports on the cancer of celebrities may have become “cues to action” to help them take action.

There have been several reports on the impact of celebrity cancer media reports on the behavior of citizens [36,37]. In Australia, mammography screening appointments increased by 40% in the two weeks following media reports of singer Kylie Minogue’s breast cancer diagnosis [38]. In the United States, Angelina Jolie’s decision to share her experience with the increased risk of breast and ovarian cancer due to BRCA1 gene mutations has improved public awareness of the disease and increased genetic testing and breast cancer screening. In particular, Angelina Jolie’s influence was reported to be related to “perceived susceptibility” and “cues to action,” which are elements of HBM [39,40].

These reports illustrate the appropriateness of interpreting the impact of personal cancer experiences and narratives on people’s emotions and behaviors based on the HBM. The findings on these effects support the validity of the interpretation that the two celebrities’ media reports were elements of “perceived susceptibility,” “perceived susceptibility,” and “cues to action” in the HBM.

### Importance of Removing Barriers

Conversely, information that provides “perceived benefits” or “self-efficacy” for screening or removes “perceived barriers” is not directly included in the celebrity cancer media reports. In contrast to the case of Angelina Jolie, where there is a direct link between her actions and the benefits of preventive behavior, there is a gap in logic between media reports of celebrity breast cancer and the benefits of screening participation. For internet users, there are few barriers to search action. However, there are significant barriers to screening participation on an entirely different level than internet searches. To participate in screening, citizens must confirm the possible dates, times, locations, and costs, make an appointment, and go to mass screening sites or hospitals. Media reports and internet search activity showed a direct relationship, while screening participation behavior showed a limited response. This suggests that information from media reports and internet searches did not remove barriers to screening participation. Some citizens who participated in screenings triggered by the media reports may not have continued to behave because they were unaware of the benefits. This finding of limited participation versus notable search activity highlights the importance of removing “perceived barriers” in the HBM component. This study’s post hoc manual search was limited to breast cancer information for 10 years. In today’s Japan, where approximately half of the population will be diagnosed with some form of cancer in their lifetime, media reports on celebrities provide citizens with many opportunities to perceive the susceptibility and severity of cancer. Nonetheless, information to remove barriers does not occur unless someone intends it. The importance of “perceived barriers” in HBM elements has long been recognized [41]. A meta-analysis of 18 communication campaigns shows that “perceived benefits” and “perceived barriers” were consistently the most robust predictors [42]. This study supports the idea that removing barriers is an essential public action to encourage healthy behaviors.

### Limitations and Strengths of This Study

There are several limitations to this study. First, there is a restriction due to the time-series analysis design. It is unknown whether specific individuals were exposed to media reports, performed search actions, or participated in screening because this is a comparison through time for the whole population. It is important to note that the results indicate only an association, and do not necessarily imply a causal relationship among celebrity news, search spikes, and screening uptake. Various real-world factors, such as concurrent public health campaigns or medical policies, could have influenced the keyword search volume, the screening participation rate, or both. Even if there is a match between exposure and outcome for an individual, it does not prove causation because confounding by unknown factors cannot be ruled out. Given this study’s data sources and design, directly evaluating causality is complex and remains an issue for future research.

Second, there is a lack of data on workplace screening. Japan’s cancer screening programs are divided into municipal screening and workplace screening. Due to incomplete legislation on workplace screening, data have not been collected and published for the entire country, and it was necessary to use only municipal screening data. In this study, to remove the effect of the lack of workplace screening data as much as possible, the age range for calculating participation rates was restricted to 60‐69 years so that retirees would represent most of the population. Due to this restriction, the generalizability of the screening data is limited. In the future, once workplace screening data becomes available, it will be necessary to include those data in the analysis to more accurately evaluate trends in screening participation rates among the working population.

Third, the coverage of Google Trends data. Given that the RSV is based on Google search data, it does not reflect the interests of populations that do not use Google or internet search. The percentage of Japanese aged 60‐69 years using the internet increased from 71.6% (60-64) and 58% (65-69) in 2009 to 90.5% (60-69) in 2019 [43]. This percentage and time change may have affected the results. Even if internet use was high enough in the age group 60s, RSV is an indicator that includes all ages and does not necessarily reflect search activity in the 60s. Furthermore, even if they use internet search, they may use a search engine other than Google. As of 2019, Google accounted for 92% of the global market share for internet search engines, 93% in Europe and 89% in North America, whereas in Japan, it was 75% [44]. While there is no doubt that Google holds the top market share in Japan, unlike Baidu in China or Yandex in Russia, its relatively lower share compared to Western regions could influence the validity of Google Trends data [45]. Further, an absolute assessment is impossible since RSV is a relative measure for a given period and search term.

Fourth, some of the methods and interpretations of this study were post hoc. In the analysis phase of this study, we found a marked increase in the RSV data for the keyword “breast cancer screening” in three specific periods. To explore background information, we performed a post hoc manual search and found that the media reports of the celebrity matched the RSV spikes. This manual search was not planned at the time the study was designed. Because these manual searches and discussions involve the arbitrariness of the researcher, careful attention should be paid to the validity of the interpretation of the results.

Furthermore, one possible reason that no joinpoints were detected for screening participation rates is that the limited number of data points may not have provided sufficient statistical power. To analyze one or more joinpoints, at least seven data points need to be observed [22]. Although this study had 11 data points for both screening and further assessment participation rates, exceeding seven, the number of data points may still have been insufficient for detecting any joinpoints.

Despite these limitations, there are strengths to this study. Google Trends, an internet search volume, is a limited source of information that directly reflects changes in the preferences and interests of the entire population over time. Media reports related to changes in internet search volume will be revealed after data analysis. Therefore, the type of study that follows participants prospectively cannot discuss what this study did. Retrospective studies that question about past exposures may cause recall or information bias due to the validity of the questionnaire. Internet search volume has none of these biases and selection biases for study enrollment, so it directly reflects citizens’ actual preferences. The screening data also have no self-reporting bias because they are actual values reported to the government by municipalities. This study design is conducive to exploring what influences public interest in cancer screening and leads to participation behavior.

### Practical Considerations and Future Implications

We found that internet search volume for “breast cancer screening” was notably associated with media reports of the celebrity’s cancer and was temporally associated with participation in screening. Although caution is needed in interpreting causal relationships, it is worth noting that the three periods in which the spike in internet search volume occurred match the media reports of the celebrities. It is reasonable to assume that media reports clearly impacted search activity. The results also showed barriers to screening participation and limitations to explaining behavior only by internet search volume. Established “model” and “effect” in behavioral medicine could describe these associations and limitations. Importantly, pragmatic data without educational intervention or questionnaire surveys supported these effects. This finding suggests that internet search volume is valuable for deciphering individuals’ behavior. Internet search volume can also help verify the effectiveness of efficacy findings confirmed by exploratory methods, such as intervention trials, with real-world data. Although it is essential to evaluate various potential biases in internet search volume, with an understanding of its limitations, using it for epidemiologic studies will continue to be beneficial and may suggest improvements in public health policy and risk communication.

### Conclusions

The public impact of celebrity cancer media reports found in this study will lead to the development of information and awareness methods to improve and sustain participation in cancer screening. A colorectal cancer awareness campaign conducted on television by Katie Couric, a well-known American television anchor, was associated with an increase in colonoscopy use [46]. The results of this study support the potential for such celebrity publicity for preventive health programs to be temporarily effective in Japan. If doing so, information on removing barriers should be included to maximize and sustain the effect.

## Abbreviations

HBM: Health Belief Model
RSV: relative search volume
